# Physiological stress response, reflex impairment and delayed mortality of white sturgeon *Acipenser transmontanus* exposed to simulated fisheries stressors

**DOI:** 10.1093/conphys/cow031

**Published:** 2016-08-26

**Authors:** Montana F. McLean, Kyle C. Hanson, Steven J. Cooke, Scott G. Hinch, David A. Patterson, Taylor L. Nettles, Matt K. Litvak, Glenn T. Crossin

**Affiliations:** 1 Department of Biology, Dalhousie University, Halifax, Nova Scotia, CanadaB3H 4R2; 2 US Fish and Wildlife Service, Abernathy Fish Technology Center, Longview, Washington, USA 98632; 3 Department of Biology, Carleton University, Ottawa, Ontario, CanadaK1S 5B6; 4 Department of Forest and Conservation Sciences, University of British Columbia, Vancouver, British Columbia, CanadaV6T 1Z4; 5 Fisheries and Oceans Canada, Cooperative Resource Management Institute, School of Resource and Environmental Management, Simon Fraser University, Vancouver, British Columbia, CanadaV5A 1S6; 6 Department of Biology, Mount Allison University, Sackville, New Brunswick, CanadaE4L 1G7

**Keywords:** Catch-and-release angling, reflex impairment, stress physiology, temperature, white sturgeon

## Abstract

White sturgeon (*Acipenser transmontanus*) are the largest freshwater fish in North America and a species exposed to widespread fishing pressure. Despite the growing interest in recreational fishing for white sturgeon, little is known about the sublethal and lethal impacts of angling on released sturgeon. In summer (July 2014, mean water temperature 15.3°C) and winter (February 2015, mean water temperature 6.6°C), captive white sturgeon (*n* = 48) were exposed to a combination of exercise and air exposure as a method of simulating an angling event. After the stressor, sturgeon were assessed for a physiological stress response, and reflex impairments were quantified to determine overall fish vitality (i.e. capacity for survival). A physiological stress response occurred in all sturgeon exposed to a fishing-related stressor, with the magnitude of the response correlated with the duration of the stressor. Moreover, the stress from exercise was more pronounced in summer, leading to higher reflex impairment scores (mean ± SEM, 0.44 ± 0.07 and 0.25 ± 0.05 in summer and winter, respectively). Reflex impairment was also correlated with lactate concentrations (e.g. physiological stress measures related to exhaustive exercise; *r* = 0.53) and recovery time (*r* = 0.76). Two mortalities occurred >24 h after the cessation of treatment in the summer. Given that natural habitats for white sturgeon can reach much higher temperatures than those presented in our study, we caution the use of this mortality estimate for a summer season, because latent mortality could be much higher when temperatures exceed 16°C. This is the first experiment investigating the physiological disturbance and reflex impairment of capture and release at two temperatures on subadult/adult white sturgeon, and the results suggest that future research needs to examine the longer term and fitness consequences of extended play and air exposure times, because these are largely unknown for wild populations.

## Abstract (precis)

- Simulated angling stress caused a physiological disturbance and reflex impairment in sub-adult/adult white sturgeon.
- Ventilation was the first reflex to become compromised, followed by orientation, mouth extension, tail grab and body flex.
- Reflex impairment indicated a compromised physiological state which correlated with the degree of the stressor (i.e. severity).
- Higher reflex impairment scores correlated with longer recovery times.
- Compromised physiological state was significantly higher in summer than winter treated fish.
- Two mortalities occurred >24 hours after the cessation of the stressor during the summer. Both individuals had all reflexes impaired upon release into the recovery tank, excess stress metabolites (i.e. lactate), and were ‘unrecovered’ following the 30 min. observation period post-release.

## Introduction

Many recreational fisheries have adopted catch-and-release (C&R) practices as a means for reducing fisheries-related mortality and conserving fish populations (Arlinghaus *et al*., 2007). The long-term success of a C&R fishery hinges on an assumption of high post-release survival (Cooke and Schramm, 2007). Research has shown that not all fish survive (reviewed by Bartholomew and Bohnsack, 2005) and that for those fish surviving, significant sublethal physiological disturbances and behavioural impairments can occur (Arlinghaus *et al*., 2007; Cooke *et al*., 2013). These effects may vary among species, life-history stages, sexes, environment (i.e. seasonal changes in water temperature) and by gear types (Cooke and Suski, 2005; Arlinghaus *et al*., 2007). For this reason, it is important not to generalize the results of C&R studies across multiple species (Cooke and Suski, 2005). The development of fisheries management plans and best practices for a target species would benefit from a better understanding of the physiological, behavioural and survival aspects of C&R impacts in different contexts, such as at different temperatures.

Reflex action mortality predictors (RAMP) have become a popular tool among researchers for the rapid assessment of fish vitality (i.e. the capacity for survival) after fisheries capture by quantitatively linking the stress response to fitness outcomes (Davis, 2005, 2007, 2010), although this technique has not yet been broadly adopted by fisheries managers. From a functional perspective, RAMP is a way of measuring the impairment of normal reflexes that provide a measure of vitality. The premise behind the reflex impairment method is that stress state and the likelihood for post-release mortality can be predicted by scoring the absence or presence of specific organismal reflexes that are normally expressed in unstressed individuals. Reflexes are defined as involuntary movements induced by a peripheral stimulus, and the suite of specific reflexes used in RAMP assessments will vary among species. Once an assessment is made, a final RAMP score is calculated and assigned to an angled fish, which serves as a proxy for compromised physiological state and whole-animal well-being (Davis, 2010; Raby *et al*., 2013). Among fishes, observable reflex impairment assessments have successfully predicted the post-release mortality of migrating coho salmon *Oncorhynchus kisutch* (Raby *et al*., 2012, 2014), sockeye salmon *Oncorhynchus nerka* (Gale *et al*., 2011, 2014), bonefish *Albula* spp. (Brownscombe *et al*., 2013) and a number of elasmobranch species (Danylchuk *et al*., 2014; Gallagher *et al*., 2014). Furthermore, reflex impairment assessments have also predicted post-release behaviour and impaired swimming performance (Brownscombe *et al*., 2013, 2014, 2015; Cooke *et al*., 2014; Szekeres *et al*., 2014). Of note, RAMP scores have also been unsuccessful in predicting post-release mortality after fisheries encounters (i.e. sockeye salmon; Robinson *et al*., 2015), further supporting the need for species- and context-specific assessments of the technique.

Understanding the physiological mechanisms that lead to reflex impairment is important for linking the sublethal effects of stress to fitness. For example, Raby *et al*. (2013) were the first to link reflex impairment scores quantitatively to physiological measures of stress and homeostasis, thereby establishing a relationship between RAMP and physiological condition. Of particular note was a significant relationship between plasma lactate (a measure of anaerobic metabolism) and RAMP score after exhaustive fisheries capture in pink salmon (*O. gorbuscha*). The authors caution, however, that lactate is not a causative agent impairing the suite of reflexes, but is instead a correlated byproduct of the complex physiological pathways more directly responsible for inducing impairment. Gallagher *et al*. (2014) also identified a correlation between lactate concentration and mortality of some shark species. However, given that clearance rates for lactate are highly variable, it is possible that the impact of high lactate concentrations on reflex impairment varies among species, and is likely to depend on when post-capture samples are collected. Although useful as an indicator of post-capture condition and reflex impairment, lactate is but one of several relevant physiological measures involved in the stress response. Each of the reflexes selected for a RAMP protocol are likely to be modulated by several different physiological pathways, and these may vary among species. It is therefore important to assess other measures of exhaustion and stress (e.g. cortisol, glucose and ions), when attempting to understand the mechanisms underlying fish vitality.

In this study, we build upon the growing body of research on reflex impairment as a potential conservation and management tool applied to important recreational fish species, and focus our efforts on the white sturgeon, *Acipenser transmontanus*. White sturgeon are exposed to fishing stressors throughout their range from California, USA, to Canada's Fraser River. Currently, white sturgeon populations exposed to fisheries in the USA are considered of least concern by the International Union for the Conservation of Nature (IUCN; Duke *et al*., 2004); however, the absence of information about pre-release stress on white sturgeon has become increasingly important for managers in the context of recreational fisheries (Z. Jackson, US Fish and Wildlife Service, Lodi, CA, USA, personal communication). Indeed, globally there is little known about how imperilled fishes respond to different C&R practices, making it difficult to determine whether C&R angling is compatible with recovery strategies (Cooke *et al*., 2016). Until 2012, all Canadian populations of white sturgeon were considered endangered; however, reassessment in 2012 resulted in the separation of populations into Designatable Units (DUs), for subsequent assessment separately. The upper Columbia, Kootenay and Upper Fraser River DUs were assessed as being endangered. The Lower Fraser River (LFR) DU was assessed by the Committee on the Status of Endangered Wildlife in Canada (COSEWIC) as being threatened; however, it is not officially recognized by the Species at Risk Act owing to its socioeconomic importance to the province of British Columbia (COSEWIC, 2012). Despite the growing popularity of recreational fishing for white sturgeon, there has been limited published work focusing on the potential impacts of acute angling stressors. Recent mark–recapture data indicate that the LFR white sturgeon population is not growing to the extent expected, and that juvenile recruitment may be an issue (Nelson *et al*., 2013). As adult white sturgeon are critical to juvenile recruitment and recovery of the population, it is essential that the effects of angling on post-release behaviour, physiology and mortality are quantified so that it can be monitored closely.

There are few to no seasonal restrictions on the recreational fishing of white sturgeon, and in Canada's Fraser River, BC, that includes angling in water temperatures ranging from <0 to >20°C in parts of their range. Therefore, this research is crucial to the understanding of the combined effects of river temperature and fishing-related stressors and how they influence the physiological status, vitality and recovery potential of recreationally angled sturgeons. Using a population of wild white sturgeon held at the US Fish and Wildlife Service, Abernathy Fish Technology Center in Longview, WA, USA, our aim was to develop, refine and validate a reflex impairment protocol by exposing sturgeon to controlled simulated fisheries capture events. We then linked the resulting reflex impairment indices (RAMP score) to stress physiology and recovery times. By doing so, we predicted that the magnitude of the physiological disturbance and reflex impairment would be correlated with treatment time, and that physiological disturbance and reflex impairment would be further modulated by water temperature. Results from these experiments allow us not only to quantify the sublethal impacts of recreational C&R stressors, but also to gauge how these might change seasonally with respect to water temperature.

## Materials and methods

A wild origin, now captive population of white sturgeon housed at the US Fish and Wildlife Service, Abernathy Fish Technology Center, Longview, WA, USA (46.226899, −123.148837) was used for our experiments. Sturgeon were maintained in two outdoor raceways (24.4 m in length × 2.4 m in width × 0.8 m in depth) with water provided at ~0.01 m^3^/s from the adjacent Abernathy Creek, a tributary to the Columbia River. Experiments ran from 25 to 29 July 2014 at an average water temperature of 15.3°C, and from 23 to 27 February 2015 at ~6.6°C.

### Experimental treatments

Forty-eight white sturgeon [24 per season; mean ± SD 109.20 ± 22.17 and 112.55 ± 18.72 cm fork length (FL) in July and February, respectively] were corralled into a sling, and allowed to thrash for a specified period of time to mimic forced exercise. Sturgeon were also partly exposed to air during this period of exercise by maintaining the sling out of water and only providing enough water to half-cover the gills. These treatments were used to elicit a stress response and push the sturgeon to their physiological limits by using a combination of stress and air exposure to simulate a fisheries interaction. Often, chasing is considered a suitable proxy for a fisheries stressor (Cooke *et al*., 2013), but given the size of our study fish and the layout of the raceway (i.e. all untreated fish in one raceway), forced thrashing in the sling provided a means of inducing a stress response while at the same time reducing the exposure of untreated fish to additional stress. In the LFR, white sturgeon angling events can last from 30 s to >2 h (median time = 5.78 min; M. F. McLean, 2015, unpubublished data).

In brief, the rectangular sling (1.84 m in length × 0.55 m in width) was constructed of tarpaulin and supported by two poles threaded through either side. To assist in capture, the sling was reinforced with a nylon hood at one end so that when corralled head first, the hood would help support the head of the sturgeon while the sides of the sling were pulled together. The poles of the sling were extended using four pieces of PVC piping so that during treatments the sling could rest across the raceway and help support the sturgeon. Individual white sturgeon were selected at random to receive a specific treatment of exercise and partial air exposure for either 0 (control), 5, 10 or 15 min, for a total of six sturgeon per treatment per season. Although we did the best to obtain immediate samples from time = 0 fish, some thrashing occurred during capture and thus our controls should be regarded as ‘handled controls’. As mentioned above, the exercise occurred in a sling with the gills partly covered in water to obtain the combined effect of partial air exposure and exercise for set periods of time. In each treatment, fish were corralled, treated, bled from the caudal vasculature and immediately checked for reflexes before being transferred to a recovery raceway.

### Reflex assessments

**Figure 1:**
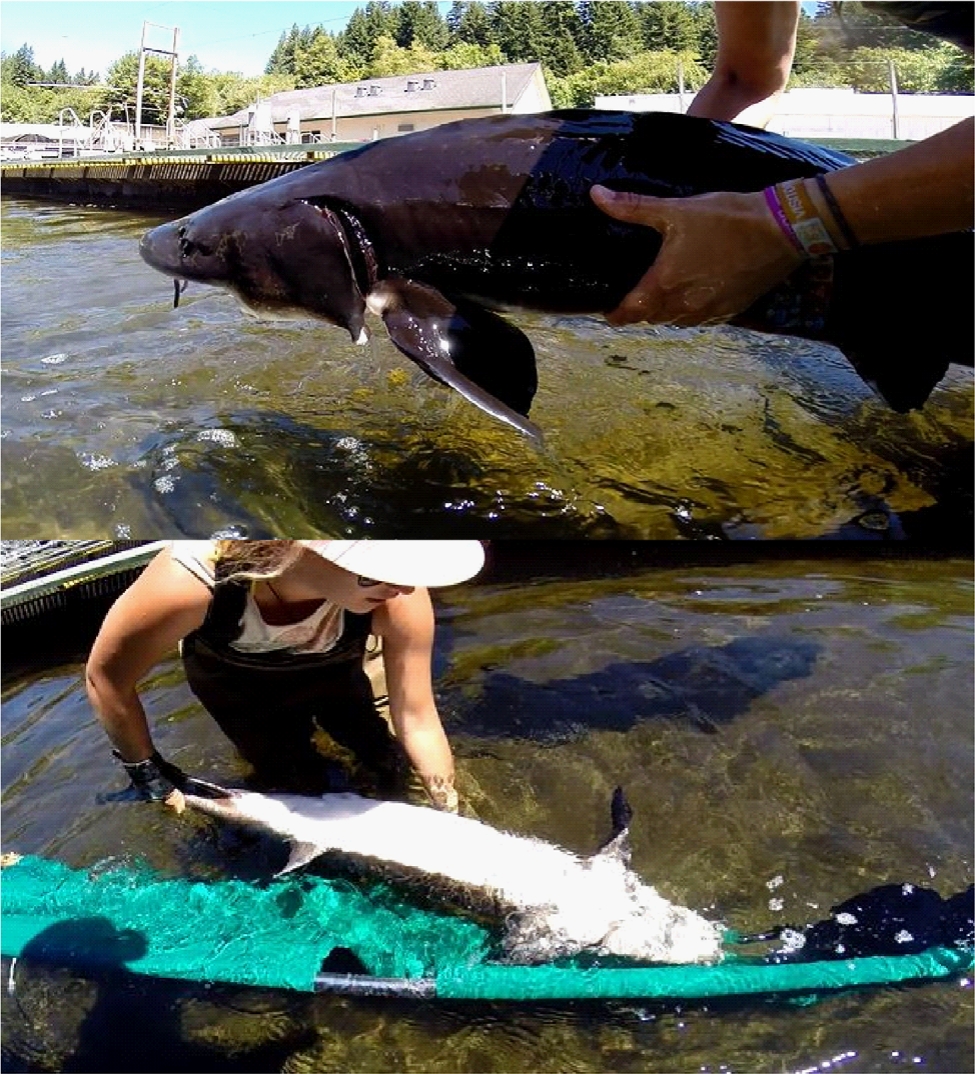
Body flex (top) and orientation (bottom) were two of five reflexes tested on white sturgeon after a combined treatment of exercise and air exposure for 0, 5, 10 or 15 min. From the reflex results for each sturgeon, a reflex action mortality predictor (RAMP) score was calculated as a simple proportion of the five measured reflexes that were impaired in an individual fish (0 = no reflexes impaired; 1 = all reflexes impaired).

A modified reflex impairment index was developed for white sturgeon based on the RAMP method previously described for other taxa (see Raby *et al*., 2013). Immediately after treatment, all sturgeon were tested for five reflexes determined to be present or absent in untreated control individuals. All reaction times were chosen based on testing these reflexes on control sturgeon. Ventilation, mouth extension, orientation, tail grab and body flex were the reflexes tested on treated white sturgeon (Fig. 1). Using a categorical assessment, reflexes were assigned a ‘0’ if the reflex was unimpaired and a ‘1’ if the reflex was impaired. Ventilation was unimpaired if the sturgeon exhibited regular ventilation for 10 s while half-submerged in the sling, as observed by watching the number of opercular movements. Mouth extension was considered unimpaired if the mouth of the sturgeon did not fully extend during a 10 s period out of water, because mouth extensions have been associated with stress events in other sturgeon species (M. F. McLean, personal observation of Atlantic sturgeon, *Acipenser oxyrinchus*). To test orientation, upon release in the recovery raceway each sturgeon was placed upside-down just below the surface. An unimpaired orientation response was noted if the sturgeon righted itself within 3 s. The tail grab response was assessed by the handler attempting to grab the caudal peduncle while the sturgeon was fully submerged in water, with an unimpaired response characterized by an immediate burst-swim response to the grab. Body flex was tested by holding the sturgeon partly out of water using two hands wrapped under the pectoral fins. If the sturgeon actively struggled free, it was characterized as an unimpaired response. All RAMP assessments were completed in <30 s. The RAMP assessments and recovery were recorded on a GoPro action video camera for each sturgeon. Some sturgeon were too vigorous to allow for complete testing of reflexes and were assigned an overall unimpaired status. This was the case for all control fish. From the reflex results for each sturgeon, a RAMP score was calculated as a simple proportion of the five measured reflexes that were impaired in an individual fish (0 = no reflexes impaired; 1 = all reflexes impaired).

### Physiology

A non-lethal blood sample was taken from the caudal vasculature of white sturgeon immediately after cessation of treatment using a 10 ml lithium heparin-coated barrel (Braun) and a 3.8 cm (1.5 inch) 18 gauge needle (Becton-Dickinson, Franklin Lakes, NJ, USA). The time to bleed was recorded to the nearest second as ‘bleed time’, and samples were immediately placed in a 50:50 ice–water slurry and processed within 30 min of collection. Glucose and lactate concentrations were measured on whole blood (10 µl) using hand-held glucose (ACCU-CHECK glucose meter; Roche Diagnostics, Basel, Switzerland) and lactate meters (Lactate Pro LT-1710 portable lactate analyser; Arkray Inc., Kyoto, Japan). These portable readers have been validated for use on fish (see Cooke *et al*., 2008) and have been used for analysis of whole blood of other sturgeon species (Atlantic sturgeon *A. oxyrinchus*, Beardsall *et al*., 2013). To determine haematocrit, a small sample of whole blood was then spun in a micro-haematocrit centrifuge (LW Scientific, Lawrenceville, GA, USA) for 5 min at 10 000***g*** (11 500 r.p.m.) to determine packed cell volume as a proportion of red cells to the total volume of the sample. The remainder of the blood sample was centrifuged (Portifuge; LW Scientific, Lawrenceville, USA) for 5 min at 3300r.p.m. to separate red cells from plasma. Plasma was transferred to cryovial tubes and then frozen and stored in a −80°C freezer at the US Fish and Wildlife Service facility until it could be analysed further.

Laboratory assays were run at the Department of Fisheries and Oceans facility in West Vancouver, British Columbia. Plasma cortisol, osmolality and Cl^−^ were quantified following the detailed instructions on the assay kits or the associated instrument instructions. In brief, hormone enzyme-linked immunosorbent assays (ELISAs) were run on plasma samples to identify cortisol titres quantitatively. According to the directions on the Neogen Corporation ELISA kit, samples and standards were run in duplicate. Inter- and intra-assay variation for cortisol were 10 and 1.8%, respectively. Osmolality was measured in plasma samples inserted into a freezing-point osmometer (model 3320; Advanced Instruments, Inc., Norwood, MA, USA). Once again, samples were run in duplicate, falling within 5 units of one another. Chloride was measured from plasma samples using a chloridometer (Haake Buchler Digital, Saddle Brook, NJ, USA). Samples were run in duplicate and fell within 3 units of one another.

### Recovery

A holistic (whole-animal assessment) approach was used to characterize recovery because all five reflexes could not be retested after the recovery period as a result of limited staffing. Anecdotally, we were interested in determining whether we could predict fate based on observational recovery, or lack thereof. Thus, sturgeon were considered fully recovered when they resumed ‘normal’ behaviours as predetermined by control fish. Any unusual behaviour was recorded up to 30 min post-release. As such, fish were considered unrecovered if normal behaviour did not ensue after the 30 min recovery period. This behaviour typically consisted of sedentary, solitary confinement to one side of the tank, and was often associated with a loss of buoyancy control in all or parts of their body. The 30 min observation period was chosen solely because of limited staffing and time, and we recognize that continued monitoring of post-release behaviour would allow us to give a better estimate of potential sublethal effects and/or delayed mortality. However, given that most wild-caught sturgeon are released immediately, we believe this observation period still offers insight to potential post-release behavioural changes.

### Statistical analysis

Physiological variables, RAMP scores and bleed and recovery times were related to sturgeon size (FL) via general linear models. To evaluate the status (i.e. vitality) of sturgeon before release into the recovery raceways, a series of two-way (season and treatment) analyses of variance (ANOVAs) were used to assess how treatment affects the physiological response and RAMP impairment scores in two seasons. A series of one-way ANOVAs were used to evaluate the relationship between blood parameters and reflex impairment. Recovery time was compared with blood physiology parameters via generalized linear regressions. One-way ANOVA was used to assess whether treatment time and/or RAMP score had an effect on recovery time. As mortality was low in our study, we tested the power of RAMP as an indicator of post-release vitality on the individuals that were observationally ‘recovered’ and ‘unrecovered’ by the end of the 30 min recovery period. Using the assumption that fish vitality is lower, and suggestive of an increased risk of delayed mortality, among individuals that did not present as fully ‘recovered’ by the end of the 30 min observational period, independent Student's *t*-test was used to compare mean RAMP scores of ‘recovered’ and ‘unrecovered’ sturgeon.

In cases of deviation from the underlying assumptions of parametric tests (normality, variance homogeneity, *P* < 0.05), continuous data were natural logarithmically (ln) transformed. In all cases, transformations successfully normalized the data (*P* > 0.05). For all comparisons, the significance was assessed at α = 0.05. *Post hoc* Tukey's HSD multiple comparison tests were used to determine significant differences (*P* ≤ 0.05). All analyses were conducted using R statistical software (R version 3.1.3; R Core Team, 2015). All data are presented as means ± SEM unless otherwise indicated.

## Results

### Reflex impairment indices

With the exception of controls, all treatments resulted in some loss of reflexes in all individuals. Ventilation was the first reflex to be affected across individuals, followed by orientation, mouth extension, tail grab and, lastly, body flex (Table 1). A proportion of sturgeon experienced reflex impairment at 5 min, except that tail grab and body flex responses remained intact. The 10 and 15 min treatments resulted in a proportion of sturgeon experiencing a loss of all five tested reflexes. All sturgeon experienced impairment of ventilation (fewer opercular beats) after the maximal treatment time of 15 min, and 90% were unable to reorient themselves (i.e. impaired orientation).

**Table 1:** Impairment of individual reflexes with increasing overall reflex impairment (RAMP score)

RAMP score (proportion)	Ventilation	Orientation	Mouth extension	Tail grab	Body flex
0.0 *n* = 10	0.00	0.00	0.00	0.00	0.00
0.2 *n* = 13	0.46	0.31	0.23	0.00	0.00
0.4 *n* = 12	1.00	0.50	0.42	0.08	0.00
0.6 *n* = 4	1.00	0.75	0.50	0.25	0.50
0.8 *n* = 6	1.00	1.00	0.17	1.00	0.86
1.00 *n* = 3	1.00	1.00	1.00	1.00	1.00

Values represent the proportion of white sturgeon with a particular reflex impaired within each level of overall impairment. For example, of the white sturgeon with overall RAMP scores of 0.4, all of them had impaired ventilation, but only 0.50 (50%) had impaired orientation.

### Effects of treatment and season on RAMP score and physiology

**Figure 2:**
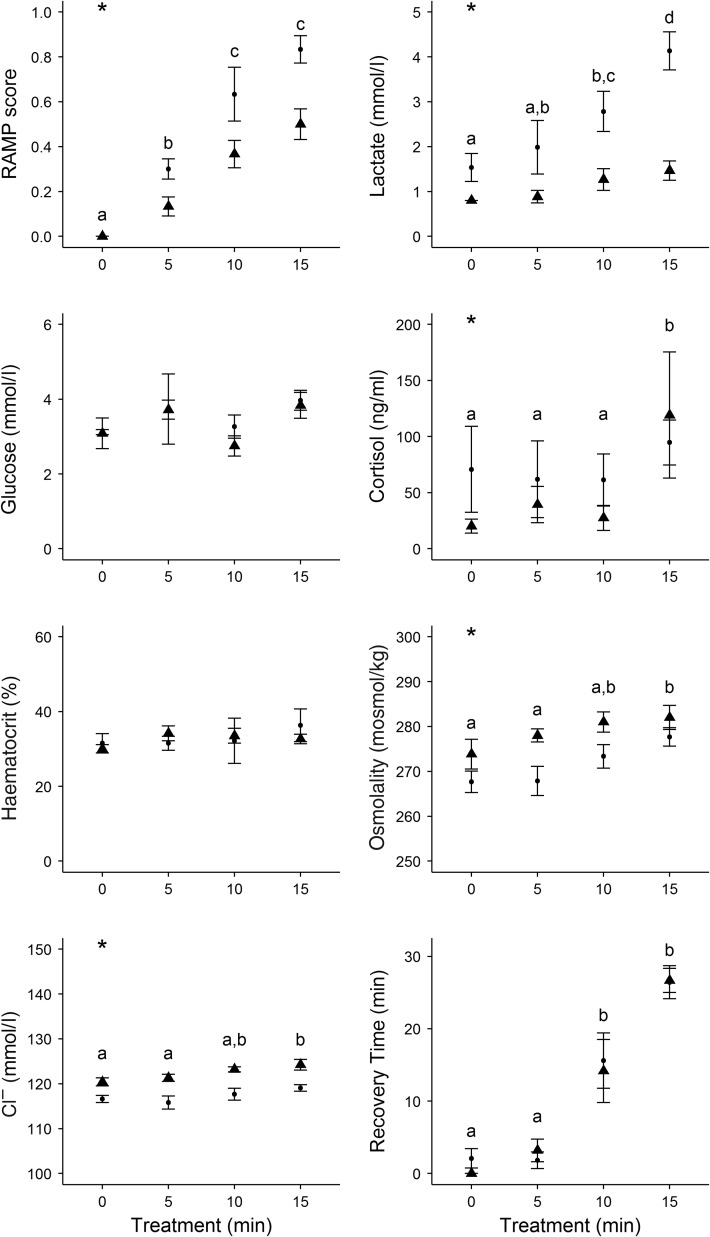
Mean ± SEM reflex action mortality predictor (RAMP) scores and physiological variables measured in white sturgeon for each of the four treatments (*n* = 12 sturgeon per treatment) in July 2014 (filled circles) and February 2015 (filled triangles). In each group, fish were corralled, treated, had a blood sample taken from the caudal vasculature, were measured to the nearest centimetre for fork length (FL) and girth and immediately checked for reflexes before being transferred to a recovery raceway. Treated sturgeon were corralled into a sling, and allowed to thrash for a specified period of time to mimic forced exercise. Sturgeon were also partly exposed to air during this period of exercise by maintaining the sling out of water and providing sufficient water only to half-cover the gills. Control sturgeon were sampled immediately. Among-group differences were assessed using a series of two-way ANOVAs (treatment and season). *Post hoc* differences among groups are indicated by dissimilar letters. *Significant difference between seasons (summer and winter) for a particular parameter.

There was no relationship between sturgeon size and indices of physiological stress, RAMP scores, handling or recovery times (*P* > 0.05). The clearest evidence of a treatment effect was seen with RAMP impairment and lactate. White sturgeon reflex impairment increased consistently with the degree of the stressor or treatment (*F*_3,40_ = 46.07, *P* < 0.001; Fig. 2). Mean RAMP score was significantly different between all treatment groups, with the exception of the RAMP scores of the 10 and 15 min groups (Fig. 2). The RAMP scores were significantly higher in summer-treated fish (*F*_1,40_ = 19.307, *P* < 0.001), but there was not a significant interaction between season and treatment group (*F*_3,40_ = 2.762, *P* = 0.054). Lactate concentrations were significantly different between treatment groups (*F*_3,40_ = 10.37, *P* < 0.001) and season (*F*_1,40_ = 42.29; *P* < 0.001), but again there was no significant interaction between treatment group and season (*F*_3,40_ = 2.04, *P* = 0.12). Specifically, a *post hoc* test revealed that the control and 5 min groups had significantly lower concentrations of plasma lactate than the 15 min treatment group (Fig. 2), and lactate was significantly higher in summer-sampled fish than winter.

**Figure 3:**
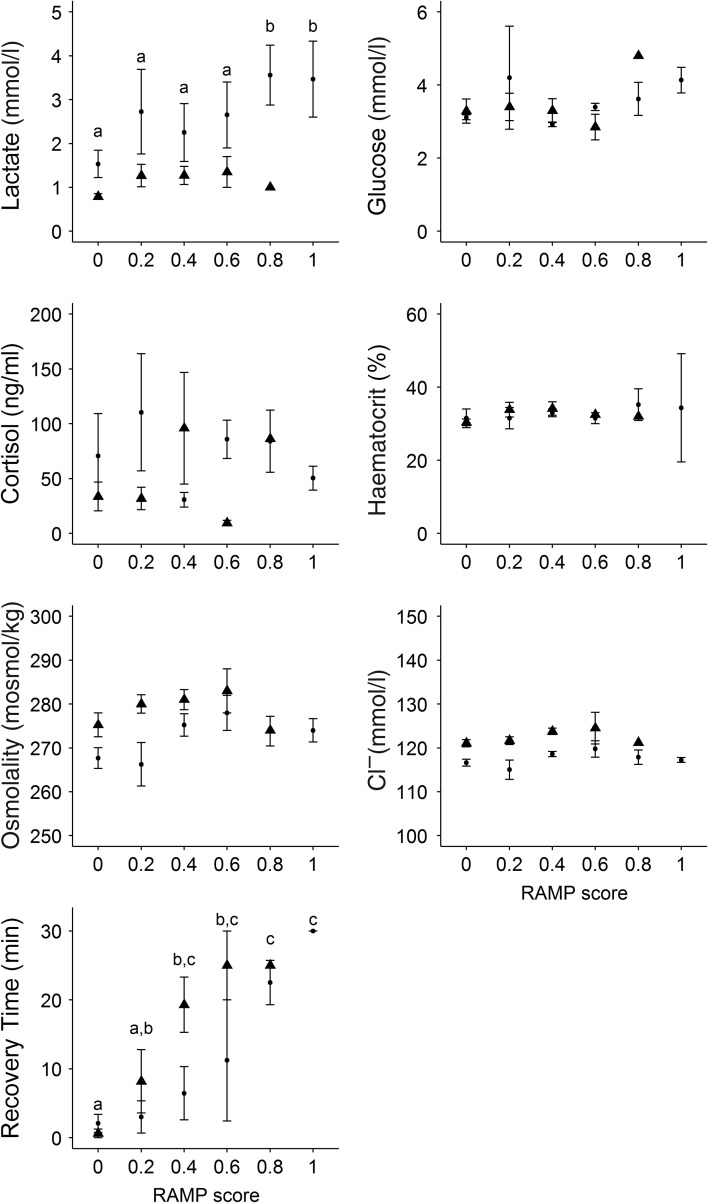
Mean ± SEM analyzed physiological parameters and recovery times for white sturgeon assessed at five different reflex action mortality predictor (RAMP) scores of overall reflex impairment in July 2014 (filled circles) and February 2015 (filled triangles). Higher scores indicate a greater proportion of impaired reflexes of the following five reflexes tested (see Fig. 2): ventilation, mouth extension, orientation, body flex and tail grab. Among-group differences were assessed via one-way ANOVA, and *post hoc* differences among groups are indicated by dissimilar letters.

Plasma cortisol was also significantly higher in the 15 min treatment group than in controls (*F*_3,40_ = 2.93, *P* = 0.04; Fig. 2) and was significantly higher in the summer sampling period (*F*_1,40_ = 4.83, *P* = 0.03). There was no significant interaction between treatment and season (*F*_3,40_ = 0.57, *P* = 0.64). Fifteen minute groups had higher glucose concentrations, but these differences were not significant (*F*_3,40_ = 2.79, *P* = 0.05; Fig. 2). Likewise, there was no significant difference in glucose concentrations between seasons (*F*_1,40_ = 0.40, *P* = 0.53) and there was no significant interaction between treatment and season (*F*_3,40_ = 0.52, *P* = 0.67). White sturgeon haematocrit ranged from 6 to 56% (mean 33%), but treatment time did not have a significant effect on the percentage of red blood cells (*F*_3,40_ = 0.50, *P* = 0.69; Fig. 3). Haematocrit was also similar between seasons (*F*_1,40_ = 0.02, *P* = 0.89), and no interaction was seen (*F*_3,40_ = 0.42, *P* = 0.74). Plasma osmolality was significantly different among treatments (*F*_3,40_ = 5.09, *P* < 0.01; Fig. 2), with a *post hoc* test revealing that individuals in the 15 min treatment groups had significantly higher osmolality than the control fish. Osmolality was also significantly higher in winter-sampled fish (*F*_1,40_ = 2.93, *P* < 0.01), but there was not a significant interaction between treatment and season (*F*_3,40_ = 0.46, *P* = 0.71). Plasma Cl^−^ was also significantly different among treatment groups (*F*_3,40_ = 4.58, *P* < 0.01; Fig. 2) and between seasons, with winter fish having significantly higher plasma Cl^**−**^ (*F*_1,40_ = 48.80, *P* < 0.01). Once again, there was not a significant interaction between treatment and season (*F*_3,40_ = 0.33, *P* = 0.80).

White sturgeon recovery time was measured up to 30 min for each individual. On average, sturgeon took 10.42 min to recover across all treatments (*n* = 46). Recovery time increased significantly with treatment time (*F*_3,42_ = 52.45, *P* < 0.01; Fig. 2), with all groups having significantly different recovery times with the exception of the control and 5 min treatments. Recovery time did not differ between seasons (*F*_1,42_ = 0.56, *P* = 0.46; Fig. 2), nor was there a significant interaction identified between treatment and season (*F*_3,42_ = 0.28, *P* = 0.84). Individuals with longer recovery times had higher concentrations of circulating lactate (*F*_1,46_ = 11.35, *P* < 0.01), higher osmolality (*F*_1,46_ = 6.85, *P* = 0.01) and higher concentrations of Cl^**−**^ (*F*_1,46_ = 4.18, *P* = 0.04). Recovery time was not, however, significantly related to plasma cortisol (*F*_1,46_ = 1.96, *P* = 0.17), glucose (*F*_1,46_ = 0.50, *P* = 0.48) or haematocrit (*F*_1,46_ = 0.24, *P* = 0.62).

### Relationship between RAMP impairment, physiology and recovery

White sturgeon with higher RAMP scores (approaching 1) had higher concentrations of lactate (*F*_5,42_ = 3.69, *P* < 0.05; Fig. 3), specifically between control groups and sturgeon with RAMP scores of 0.8 and 1. Natural logarithmically transformed plasma cortisol and blood glucose concentrations were not significantly different among RAMP scores (*F*_5,42_ = 0.59 and *F*_5,42_ = 1.18, *P* > 0.05, respectively; Fig. 3). Likewise, haematocrit, osmolality and Cl^**−**^ were not significantly different among RAMP scores (*F*_5,42_ = 2.37, *F*_5,42_ = 0.84 and *F*_5,42_ = 1.66, *P* > 0.05, respectively; Fig. 3). Individuals with higher RAMP scores did take significantly longer to recover (*F*_5,42_ = 11.90, *P* < 0.01; Fig. 3). Specifically, there were significant differences in recovery time between individuals with impairment scores of 0 and 0.4, 0.6, 0.8 and 1, as well as between 0.2, 0.8 and 1.0.

### Prediction of fate and delayed mortality

Of 48 treated individuals, nine were considered ‘unrecovered’ by the end of the 30 min observational period (*n* = 5 in summer and 4 in winter). All unrecovered fish were from the longer treatment groups (*n* = 7 from 15 min and 2 from 10 min) and had significantly higher reflex impairment scores (mean 0.69, SD 0.30) than recovered fish (mean 0.27, SD 0.26; *t*(11.02) = 3.87, *P* < 0.01).

Of the nine unrecovered fish, only two mortalities occurred after 10 and 15 min treatments in the summer sampling period (July 2014). One mortality occurred 48 h after cessation of the treatment and the other, <72 h after treatment. The RAMP scores for both fish were 1, indicating that all reflexes were impaired at the time of release (Table 2). Both mortalities revealed opposing but extreme haematocrit profiles of 6 and 56%, along with upper-range concentrations of circulating lactate and mid-range cortisol, glucose and osmolality (Table 2). One individual presented with a number of skin lesions suggestive of a potential pre-treatment illness.

**Table 2:** Size, capture and handling times, physiological parameters and reflex action mortality predictor (RAMP) scores for white sturgeon after simulated capture treatments in captivity in July 2014 (summer) and February 2015 (winter)

Measurement	No mortality (*n* = 46)				Mortality (*n* = 2)	
	Summer		Winter		Summer only	
	Mean (SD)	Range	Mean (SD)	Range	Mean (SD)	Range
Fork length (cm)	106.8 (21.7)	63.5–170.2	112.6 (18.7)	72.5–149.9	117.1 (32.0)	94.5–139.7
Girth (cm)	42.0 (7.6)	30.5–61.0	46.4 (8.3)	33.0–62.2	56.4 (2.2)	54.9–57.9
Treatment (min)	–	0.0–15.0	–	0.0–15.0	–	10.0–15.0
Bleed time (min)	2.6 (1.3)	1.2–6.9	1.9 (0.7)	1.0–3.3	3.0 (1.4)	2.0–4.0
Lactate (mmol/l)	2.5 (1.5)	0.8–5.3	1.1 (0.5)	0.8–2.4	3.9 (1.8)	2.6–5.2
Glucose (mmol/l)	3.4 (1.2)	2.4–8.4	3.3 (0.9)	1.6–4.8	4.5 (0.4)	4.2–4.7
Cortisol (ng/ml)	73.2 (72.8)	13.47–261.4	51.5 (79.4)	7.2–383.5	61.2 (4.4)	58.1–64.3
Haematocrit (%)	33.0 (6.2)	22.0–52.0	32.6 (4.1)	25.0–43.0	31.0 (35.4)	6.0–56.0
Osmolality (mosmol/kg)	271.2 (7.5)	256.0–283.0	278.7 (6.6)	261.0–289.0	276.5 (2.1)	275.0–278.0
Chloride (mmol/l)	118.0 (3.0)	110.1–121.6	122.3 (2.6)	117.9–128.1	117.2 (1.3)	116.2–118.1
RAMP score (proportion)	0.4 (0.3)	0.0–1.0	0.3 (0.2)	0.0–0.8	1.0 (0.0)	–
Recovery time (min)	9.8 (10.8)	0.0–30.0	11.0 (12.1)	0.0–30.0	–	–

Both mortalities occurred in July 2014.

## Discussion

### Reflex impairment indices

Our study demonstrates that reflex impairment (RAMP) indices are a promising tool to predict post-release vitality in white sturgeon exposed to acute fisheries encounters, such as an angling event. The reflexes used in our RAMP protocol were chosen so that multiple neurological and/or muscle pathways underlying the overall stress response were tested. What we found was that sturgeon exposed to fishing-related stressors had higher RAMP scores and took significantly longer to recover than control fish. The relationship between reflex impairment and stressor intensity (i.e. fishery-related treatment) indicates that sturgeon are undergoing whole-animal (or tertiary) responses to varying degrees of capture stress.

Reflex impairment indicators were surprisingly sensitive to fisheries stressors. Control fish had all reflexes intact, whereas multiple reflexes were absent after fish were treated. For white sturgeon, ventilation, mouth extension and orientation were typically the first reflexes affected by treatment, followed by body flex and tail grab. Indeed, ventilation and orientation are commonly affected fairly quickly following exposure to a stressor across fish taxa (Raby *et al*., 2013; sockeye salmon, Gale *et al*., 2011, 2014). Body flex and tail grab reflexes were absent in most white sturgeon exposed to the longer treatments (10 and 15 min). This lack of body tone and/or ability to respond to a physical stimulus is likely to be a result of white muscle exhaustion (i.e. increased lactate; Raby *et al*., 2013) and has been identified in other stressed fishes (Raby *et al*., 2013; Brownscombe *et al*., 2013, 2014; Szekeres *et al*., 2014). Interestingly, a number of salmon species subjected to fisheries stressors lost their ability to respond to physical stimulus almost immediately (sockeye, Gale *et al*., 2011, 2014; coho, Raby *et al*., 2013), whereas white sturgeon were fairly resilient to the loss of those reflexes until they were exposed to the longer treatments. This difference in reflex responsiveness is likely to be a result of the reduced anaerobic stress response in sturgeon when compared with teleosts (Kieffer *et al*., 2001). Anterior positive buoyancy was also recognized in stressed white sturgeon. Sturgeon have open (physostomous) swim bladders that connect directly to the oesophagus. Swim bladder volume (i.e. buoyancy) is therefore regulated by gulping or expelling gas (Brown *et al*., 2013). The loss of equilibrium in white sturgeon could be explained in part by the loss of controlled regulation of the swim bladder, subsequently causing the swim bladder to inflate and create positive buoyancy. Indeed, neutral buoyancy was regained after the expulsion of air; a process that took longer for individuals that experienced longer treatments.

### Physiological findings and reflex impairment

The underlying assumption of reflex impairment is that it has a basis in the physiological stress response (Davis, 2010). Greater reflex impairment (i.e. higher RAMP scores) was indeed associated with physiological exhaustion (i.e. increased lactate), suggesting that RAMP scores can indicate or predict an alteration in physiological condition in angled sturgeon. This movement away from homeostasis is subsequently a predictor of overall vitality or the capacity for survival. The direct measurement of reflex impairment after the stressor suggests that physiological disturbances that manifest quickly are likely to be causes of impairment, further supporting the tight association between lactate concentrations and reflex impairment indices in this study and a number of others (i.e. Raby *et al*., 2013; Brownscombe *et al*., 2013; McArley and Herbert, 2014; Gale *et al*., 2014). Latent physiological changes, such as increases in circulating plasma cortisol, are therefore unlikely to be direct causes of reflex impairment (McArley and Herbert, 2014), and this may explain why we did not find a relationship between other tested indices of physiological stress and RAMP scores.

In addition to examining the relationship between RAMP score and physiology, the individual physiological parameters were compared with treatment time. As expected, sturgeon demonstrated a stress response that was related to the magnitude of the applied stressor. In particular, circulating lactate, glucose, plasma osmolality and Cl^**−**^ were significantly higher in individuals that were treated for longer (Fig. 2). Similar physiological profiles have been demonstrated in sturgeons exposed to varying degrees of stressors (Semenkova *et al*., 1999; Kieffer *et al*., 2001; Bayunova *et al*., 2002; Baker *et al*., 2005; Zuccarelli *et al*., 2008; Beardsall *et al*., 2013). There was not a significant difference in cortisol between treatments, but given that cortisol has been shown to peak in white sturgeon ~30 min after exposure to air (Zuccarelli *et al*., 2008), it is likely that maximal cortisol concentrations were not achieved as a result of blood sampling immediately after the stressor. Likewise, a secondary stress response was elicited in sturgeon exposed to longer treatments, as indicated by elevated levels of circulating glucose, lactate, haematocrit, plasma osmolality and Cl^**−**^. Glucose was higher in 15 min treatment groups than in control fish, but the difference was not significant. The effects of acute stressors on blood glucose vary between sturgeon species from moderate to completely absent. It is possible that low glucose concentrations coincide with a lower metabolic rate in sturgeon; an advantageous characteristic that may be used to compensate for periods of hypoxia in their natural environment (Baker *et al*., 2005). It is also possible that changes in glucose were delayed, given that the cortisol response was also not evident. Increased cortisol should stimulate higher glucose concentrations (Wendelaar Bonga, 1997), potentially indicating an issue with the sampling time immediately after the cessation of treatment.

Anaerobic metabolism was activated in sturgeon, as demonstrated by the increase in circulating lactate. Likewise, an increase in haematocrit is a common response to higher oxygen demands, and increases in haematocrit have been noted in other sturgeon exposed to acute stressors (Kieffer *et al*., 2011). Although haematocrit was not significantly different among treatment groups, sturgeon treated for longer showed markedly higher haematocrit than control fish. Lastly, the increase in osmolality and Cl^**−**^ demonstrates that osmotic and ionic redistribution occurred during the stress response. Taken together, these results suggest that anaerobic metabolism does contribute to the energy budget of white sturgeon exposed to fisheries-related stressors.

Allometric differences in physiological and reflex responses to stress were not explored in this study but should be considered in future studies. Larger body size has been connected to greater physiological disturbance in other fish species. For example, after exposure to exhaustive exercise, large largemouth bass (*Micropterus salmonids*) exhibited elevated concentrations of plasma glucose and sodium relative to small fish, and they required additional time to clear the metabolites (Gingerich and Suski, 2012). These results indicate that smaller fish have an improved ability to recover from disturbances, suggesting that more work needs to be done on larger white sturgeon because our results could be underestimating the potential effects on those size classes. Between 2013 and 2016, M.F.M. and colleagues angled and tagged white sturgeon in the Lower Fraser River at a mean size of 192 cm FL (M. F. McLean, unpublished data). Therefore, although much larger fish (>3 m; M. F. McLean, unpublished data) can be caught, the size class from the present study (110 cm FL) is comparable to the mean sizes of white sturgeon caught in the wild C&R fishery, suggesting that the results presented in this study are representative of the majority of fish caught.

### Proximate causes of delayed mortality

Reflex impairment indices are commonly used to predict delayed mortality in aquatic organisms where post-release monitoring is difficult (Davis, 2005, 2007, 2010; Raby *et al*., 2013; Cooke *et al*., 2014). Indeed, the two mortalities in our study occurred >24 h after cessation of the stressor, both individuals showed impairment across all reflexes at the time of release into recovery raceways, and both failed to recover during the 30 min post-release recovery observation period. The proximate cause of delayed mortality in this study is unknown. One prediction is that lower levels of physiological disturbance and, subsequently, lower reflex impairment during the capture and handling of individuals can promote better survival upon release, particularly during warm temperatures. Indeed, the two mortalities occurred in fish exposed to longer treatment times (10 and 15 min) in the summer sampling period (July; mean temperature 15.6°C), and both fish had upper-range levels of physiological indicators of stress (i.e. lactate) as well as overall RAMP impairment scores of 1 (all reflexes impaired). In fact, reflex impairment had a stronger association with delayed mortality than the physiological metrics, as other surviving fish had similar physiological profiles to the deceased fish but lower reflex impairment scores. One exception was the extreme haematocrit profile of the two dead individuals (6 and 56%; Table 2). During the stress response, there is an increased oxygen demand for the tissues that can result in the rapid release of stored red blood cells into circulation. This results in a measure of increased percentage of red blood cells, or haematocrit; a haematological profile that is common in stressed fishes (Barton, 2002). Baseline haematocrit values for white sturgeon have been reported as ~30% (Baker and Brauner, 2011), which suggests that the individual with haematocrit of 6% was in an anaemic state before the treatment stressor and likely to be predisposed to an increase risk of delayed mortality. Given that there were so few mortalities in our study, it is difficult to identify the lethal thresholds for white sturgeon using indicators of excess stress metabolites or anaerobic acid–base/ionic imbalances. However, the significant relationship we found between the lactate concentration and reflex impairment suggests that excess stress metabolites may contribute to delayed mortality, as has been shown in studies on other species (Gale *et al*., 2014).

It is important to note that it was not the aim of this study to produce accurate mortality estimates for use in C&R fisheries, but rather to explore the use of RAMP on a sturgeon species frequently angled in the wild. We recognize the subjectivity of a whole-animal assessment and categorization; however, given the statistically significant difference in RAMP scores of observationally ‘recovered’ and ‘unrecovered’ sturgeon, we suggest that RAMP is an effective tool for predicting a lowered state of vitality post-release and that this suggests a continuum to an increased risk of delayed mortality.

### Effects of temperature

Warmer water temperatures affected the stress response of white sturgeon, as evidenced by some of the plasma variables and reflex impairment indices. In particular, cortisol and lactate were significantly higher in summer-treated sturgeon, whereas chloride and osmolality were significantly lower after each treatment. Previous studies have demonstrated that water temperature modifies the physiological stress response in green sturgeon, *Acipenser medirostris* (Lankford *et al*., 2003), and Atlantic sturgeon *A. oxyrinchus* (Kieffer *et al*., 2011), which may explain some of the variation between seasonal physiology values. For example, Atlantic sturgeon exposed to severe (<10 mmHg) hypoxia for 1 h at 5 or 15°C had increased lactate concentrations compared with individuals in normoxic conditions, indicating an increase in anaerobic metabolism (Kieffer *et al*., 2011). Additionally, there was a significant increase in plasma glucose concentrations solely at the cooler temperature (5°C), suggesting that fuel demands differ for Atlantic sturgeon at different temperatures (Kieffer *et al*., 2011). Lankford *et al*. (2003) noted that the rate of synthesis of cortisol was delayed in green sturgeon acclimated to 11°C compared with those acclimated to 19°C, probably because of a temperature-dependent reduction in enzymatic activity resulting in decreased synthesis rates of cortisol. This is important because, although we saw a dampened cortisol response in winter-treated fish, it is possible that peak concentrations were overlooked owing to the slower rate of synthesis. Interestingly, they also found a slower rate of plasma cortisol clearance at cooler water temperatures; an important finding when considering post-release recovery of white sturgeon in wild populations.

Higher resting plasma lactate was also noted in green sturgeon acclimated to higher water temperatures (Lankford *et al*., 2003). Sturgeon occupy a range of thermal regimes, so the impact of water temperature on fish performance is expected to differ from that on stenothermal species, on which many studies of temperature effects are based. Upper thermal tolerances of green sturgeon have been identified as ~33°C, above which normal ventilatory function is inhibited. Exposure to near-lethal temperatures caused an increase in haematocrit and plasma osmolality in green sturgeon, probably as a result of the elevated metabolic demands of temperature increase and the subsequent increase in the osmotic gradient across the gill (Lankford *et al*., 2003). Higher water temperatures (19°C) have also been linked to elevated plasma chloride and lactate in Pacific salmon, suggesting a disturbance in osmoregulatory homeostasis (Jeffries *et al*., 2012). In fact, ~70% of studies examining the effects of fisheries C&R stress at different temperatures have found a positive relationship between water temperature, indices of stress and mortality (see Gale *et al*., 2013 for full review; Gale *et al*., 2011, 2014; Robinson *et al*., 2013; Raby *et al*., 2015). It is unknown why Cl^−^ and osmolality were significantly lower in summer-treated fish, but one hypothesis is that osmoregulatory impairment may be related to the higher impact of the stressor on particular reflexes, such as ventilation, at warmer temperatures. Sturgeon reflex impairment was indeed dependent on temperature, with higher temperatures causing greater reflex impairment at each level of treatment. Higher temperatures generally coincide with higher resting metabolic rates and higher oxygen demands. Our control sturgeon did indeed have higher resting ventilation rates in the summer sampling period. Although ventilation was impaired in both seasons, it became impaired sooner in summer-treated sturgeon. The impaired ventilation probably translates to reduced gas exchange and oxygen uptake, resulting in an inability to recover oxygen debt from anaerobic exercise quickly, which is one potential explanation for the summer mortalities.

### Conclusions and management implications

Our study highlights the importance of examining whole-animal changes when characterizing sturgeon stress responses to fisheries-related stressors. Furthermore, the relationship between stress physiology and reflex impairment in white sturgeon suggests that RAMP could provide a simple and inexpensive evaluation of fish vitality after exposure to fisheries-related stressors, and could be used without the use of more invasive research methods on sturgeon (e.g. blood physiology). The difference in physiology and reflex impairment in winter- and summer-sampled sturgeon highlights the importance of considering management plans for wild fisheries, because the same stressor intensity can have varying impacts at different temperatures. Given that the water temperatures in our study were mild in comparison to summer temperatures that white sturgeon often experience in the wild (i.e. maximal LFR temperature mid-July 2015 = 21.20°C; data provided by the Ministry of Forests Lands and Natural Resource Operations of British Columbia), we suggest that future work should investigate the physiological stress response, reflex impairment and recovery, in wild fish at extremely high temperatures. Additionally, the strong correlation between reflex impairment and recovery time suggests that reducing play time and handling could benefit wild sturgeon by reducing the time it takes them to recover.

The combination of exercise stress and partial air exposure typical of angling was physiologically demanding for white sturgeon in our study, but it is not likely to be a direct cause of mortality if the duration is <15 min at temperatures between 6 and 16°C. Our mortality estimates should be interpreted carefully in relation to wild C&R fisheries, because angling events often last much longer than what was explored in this study, and the events are often coupled with angler experience, gear type, temperature and species (Arlinghaus *et al*., 2007). The individual roles of air exposure and exercise were not parsed out in this study; however, this is a topic that requires further attention because each stressor may not have an equal impact on the stress physiology and impairment of white sturgeon.

Reflex action mortality predictors proved to be an effective measure of sublethal stress and are therefore a potentially useful tool for assessing the release condition of angled white sturgeon. In particular, our RAMP methodology should be applied in future studies where the impacts of extended angling events and strenuous exercise in wild white sturgeon are evaluated *in situ*, e.g. the Fraser River recreational sturgeon fishery. The changes in stress physiology and reflex impairment suggest to us that future work needs to examine the longer-term fitness consequences (i.e. growth impairment and reproductive impacts) of extended play and air exposure times, because these are largely unknown for wild populations. In particular, future research should examine the impacts of extended periods of strenuous exercise in wild white sturgeon, because the methods used in our study do not fully reproduce the intensity and/or duration of a real angling event. Furthermore, allometric and sex differences in the physiological stress response of white sturgeon require attention.
